# Gut microbiota alterations in children and their relationship with primary immune thrombocytopenia

**DOI:** 10.3389/fped.2023.1213607

**Published:** 2023-06-21

**Authors:** Xiangyu Li, Minna Zhang, Le He, Jingfang Zhou, Peng Shen, Weijie Dai, Xiaozhong Yang, Yufang Yuan, Haiyan Zhu, Honggang Wang

**Affiliations:** ^1^ Department of Gastroenterology, The Affiliated Huaian No. 1 People’s Hospital of Nanjing Medical University, Huaian, China; ^2^ Pediatrician Department, The Affiliated Huaian No. 1 People's Hospital of Nanjing Medical University, Huaian, China

**Keywords:** primary immune thrombocytopenia, children, gut microbiota, abundance, diversity, antiplatelet antibodies

## Abstract

**Introduction:**

Gut microbiota reportedly play a critical role in some autoimmune diseases by maintaining immune homeostasis. Only a few studies have examined the correlation between gut microbiota and the onset of primary immune thrombocytopenia (ITP), especially in children. The purpose of this study was to investigate changes in the composition and diversity of the fecal microbiota of children with ITP, as well as the correlation between such microbiota and the onset of ITP.

**Methods:**

Twenty-five children newly diagnosed with ITP and 16 healthy volunteers (controls) were selected for the study. Fresh stool samples were collected to identify changes in the composition and diversity of gut microbiota as well as for potential correlation analysis.

**Results:**

In ITP patients, the phyla that were most frequently encountered were Firmicutes (54.3%), followed by Actinobacteria (19.79%), Bacteriodetes (16.06%), and Proteobacteria (8.75%). The phyla that were predominantly found in the controls were, Firmicutes (45.84%), Actinobacteria (40.15%), Bacteriodetes (3.42%), and Proteobacteria (10.23%). Compared with those of the controls, the proportions of Firmicutes and Bacteriodetes in the gut microbiota of ITP patients were increased while the proportions of Actinobacteria and Proteobacteria were decreased. Furthermore, gut microbiota in ITP patients varied by age group, showed specific changes in diversity, and were correlated with antiplatelet antibodies. IgG levels were significantly positively correlated with Bacteroides (*P*<0.01).

**Conclusions:**

The gut microbiota of children with ITP are imbalanced, as shown by the increase in Bacteroidetes, which was positively correlated with IgG. Thus gut microbiota may contribute to ITP pathogenesis via IgG.

**Clinical Trial Registration:**

The clinical trial were registered and approved by the Institutional Review Committee of The Affiliated Huaian No.1 People’s Hospital of Nanjing Medical University. Ethics number KY-2023-106-01.

## 1. Introduction

Primary immune thrombocytopenia (ITP) is an acquired autoimmune disease underlined by a variety of etiologies. It is a common immune-mediated bleeding disease in children with an annual incidence of approximately (5–10)/100,000. It is characterized by increased platelet destruction and impaired platelet production, resulting in a temporary or persistent decrease in platelet counts and disordered development and maturation of bone marrow megakaryocytes (1, 2). The main clinical manifestations include skin and mucous membrane bleeding, and epistaxis. Visceral bleeding may occur (3, 4). Less than 10% of ITP patients experience severe bleeding, including 0.1%–0.9% with life-threatening intracranial hemorrhage (5). Early treatment does not reduce the incidence of chronic ITP but helps recover platelets in a short time, which reduces the occurrence of serious bleeding events (6).

Gut microbiota plays a vital role in maintaining homeostasis of the immune system. Several studies have contended that microbes may play an important role in the development of autoimmune diseases (7–9). Clinical studies aimed at investigating the association between gut microbiota and adult ITP susceptibility are currently ongoing (10–12), Zhang et al. (10), found that the gut microbiota and the metabolome of patients with ITP displayed metabolic abnormalities. There was a strong negative correlation between platelet counts and intestinal microbiota, as well as between platelet counts and intestinal metabolites. Liu et al. (11), showed that platelet activation in patients with ITP was associated with their gut microbiota, and proposed that ITP may compensate for platelet activation in some way. Wang et al. (12), found that corticosteroid therapy affected the intestinal microbiota of patients with ITP, as evidenced by the differences between the gut microbiota of patients with ITP who received corticosteroid treatment and those who did not. The findings of the above studies, indicate that gut microbiota may be associated with the incidence of ITP. In this study, we aimed to lay the foundation for further research on gut microbiota and corticosteroid resistance in ITP.

In this study, we analyzed the changes in the composition and diversity of the gut microbiota of children with ITP, as well as their correlation with the occurrence of diseases, in order to explore the characteristics of gut microbiota in children with ITP and its role in the etiology of this illness.

## 2. Materials and methods

### 2.1. Patient and sample collection

From June 2019 to August 2020, we conducted a study of pediatric patients newly diagnosed with ITP according to the ITP diagnostic criteria proposed by the Clinical Guidelines. Diagnostic essentials (13) were as follows: (i) At least two routine blood examinations that showed a decrease in the platelet count (<100 × 10^9^/L), despite the morphology of blood cells in peripheral blood smears being normal; (ii) no enlargement of spleen generally; (iii) bone marrow examination showed increased megakaryocytes or normal with maturity disorder; and (iv) exclusion of other secondary thrombocytopenias. Twenty-five patients with newly diagnosed ITP were enrolled in this study (group P). Sixteen sex- and age-matched healthy volunteers were recruited as controls (group C). Fresh fecal samples were taken from the twenty-five patients with ITP before treatment, and from the 16 healthy controls.

The study was approved by the Institutional Review Committee of The Affiliated Huaian No. 1 People's Hospital of Nanjing Medical University. A written informed consent was obtained from each patient and volunteer guardian prior to enrollment.

### 2.2. Sample preparation

A single-use sterility kit was used according to the manufacturer's instructions. Stool samples were collected in the morning on empty stomachs. For further testing, the stool samples were stored at −80°C in the refrigerator.

### 2.3. Gut microbiota analysis

Microbial community genomic DNA was extracted from fecal samples using an E.Z.N.A.® soil DNA Kit (Omega Bio-tek, Norcross, GA, U.S.), according to the manufacturer's instructions. The DNA extract was analyzed on 1% agarose gel, and DNA concentration and purity were determined using a NanoDrop 2000 UV-vis spectrophotometer (Thermo Scientific, Wilmington, USA). The hypervariable region V3–V4 of the bacterial *16S rRNA* gene were amplified using primer pairs 338F (5′-ACTCCTACGGGAGGCAGCAG-3′) and 806R (5′-GGACTACHVGGGTWTCTAAT-3′) by an ABI GeneAmp® 9700 PCR thermocycler (ABI, CA, USA). PCR amplification of 16S rRNA was performed as follows: initial denaturation at 95°C for 3 min; 27 cycles of denaturing at 95°C for 30 s; annealing at 55°C for 30 s and extension at 72°C for 45 s; a single extension at 72°C for 10 min, ending at 4°C. The PCR product was extracted from 2% agarose gel and purified using an AxyPrep DNA Gel Extraction Kit (Axygen Biosciences, Union City, CA, USA) according to the manufacturer's instructions and quantified using Quantus™ Fluorometer (Promega, USA).

Operational taxonomic units (OTUs) with a 97% similarity cutoff were clustered using UPARSE version 7.1, and the chimeric sequences were identified and removed. The taxonomy of each representative OTU sequence was analyzed using the RDP Classifier algorithm 2 against the 16S rRNA database (14).

### 2.4. Calculation of diversity and richness index

Percent relative abundance (%) was used to represent the relative abundance of operational taxonomic units (OTUs) and species (15). Alpha diversity of OTUs were estimated using Chao 1 and Shannon indices (16). Chao1 is an index of bacterial species used to estimate the number of OTUs in a community. The formula for this index is Schao1 = Sobs + *n*1(*n*1 − 1)/2(*n*2 + 1), where Sobs is the number of observed OTUs, *n*1 is the number of OTUs with only one sequence, and *n*2 is the number of OTUs with only two sequences. Shannon's index (H) is used to estimate the microbial diversity in a sample, and it is estimated using the following formula: H = −Σ(Pi) (ln Pi), where Pi is the proportion of individuals belonging to species in the sample. The larger the Shannon value, the higher the community diversity.

### 2.5. Statistical methods

Parametric data are expressed as mean ± standard deviation. Data analysis procedures were performed using SPSS statistical software package (version 22.0). *P* values <0.05 were considered statistically significant.

## 3. Results

### 3.1. Clinical features of the study population

Twenty-five newly diagnosed ITP patients (Group P; 13 boys, 12 girls; age range, 11 months–10 years; average 46.5 months) were enrolled. In order to establish controls, sixteen healthy children of matching gender and age were recruited [group C (control group); 9 boys, 7 girls; age range, 1–10 years old; average 47 months]. Group P was divided into three subgroups: P1 (1–3 years old); P2 (3–6 years old); and P3 (>6 years). Similarly, Group C was divided into three subgroups corresponding to the ages of P1, P2 and P3, as C1, C2, and C3. In addition, stool swab samples were taken from 25 patients with ITP before treatment and the 16 controls for analysis. The patients' age at onset, disease duration, time to first discovery of thrombocytopenia, first platelet count, and time taken by platelets to return to normal were recorded (Table 1). None of the participants had any serious diseases or health problems. There were no significant gender or age differences between the groups (Table 2).

**Table 1:** Clinical features of ITP patients.

	P
Ages (month)	46.5 ± 32.3
Sex (M/F)	13/12
Platelet count (×10^9^/L)	16.2 ± 11.5
Disease duration (days)	4.8 ± 1.6
Time to first discovery of thrombocytopenia (days)	13.4 ± 30.7
The time for platelets to return to normal (days)	4.8 ± 1.6

P, P group/disease group.

**Table 2:** Clinical characteristics of the study population.

	P	C	*p*	P1	C1	*p*	P2	C2	*p*	P3	C3	*p*
Age (month)	46.5	47	0.942	17	30.56	0.227	38.68	47	0.521	77	92	0.507
Sex			0.522			0.455			0.231			0.53
Boy	13	9		6	3		3	4		4	3	
Girl	12	7		3	3		6	2		3	1	

P, P group/disease group; C, C group/healthy control group; *p, p values*.

### 3.2. Differences of gut microbiota between group P and C

**Figure 1:**
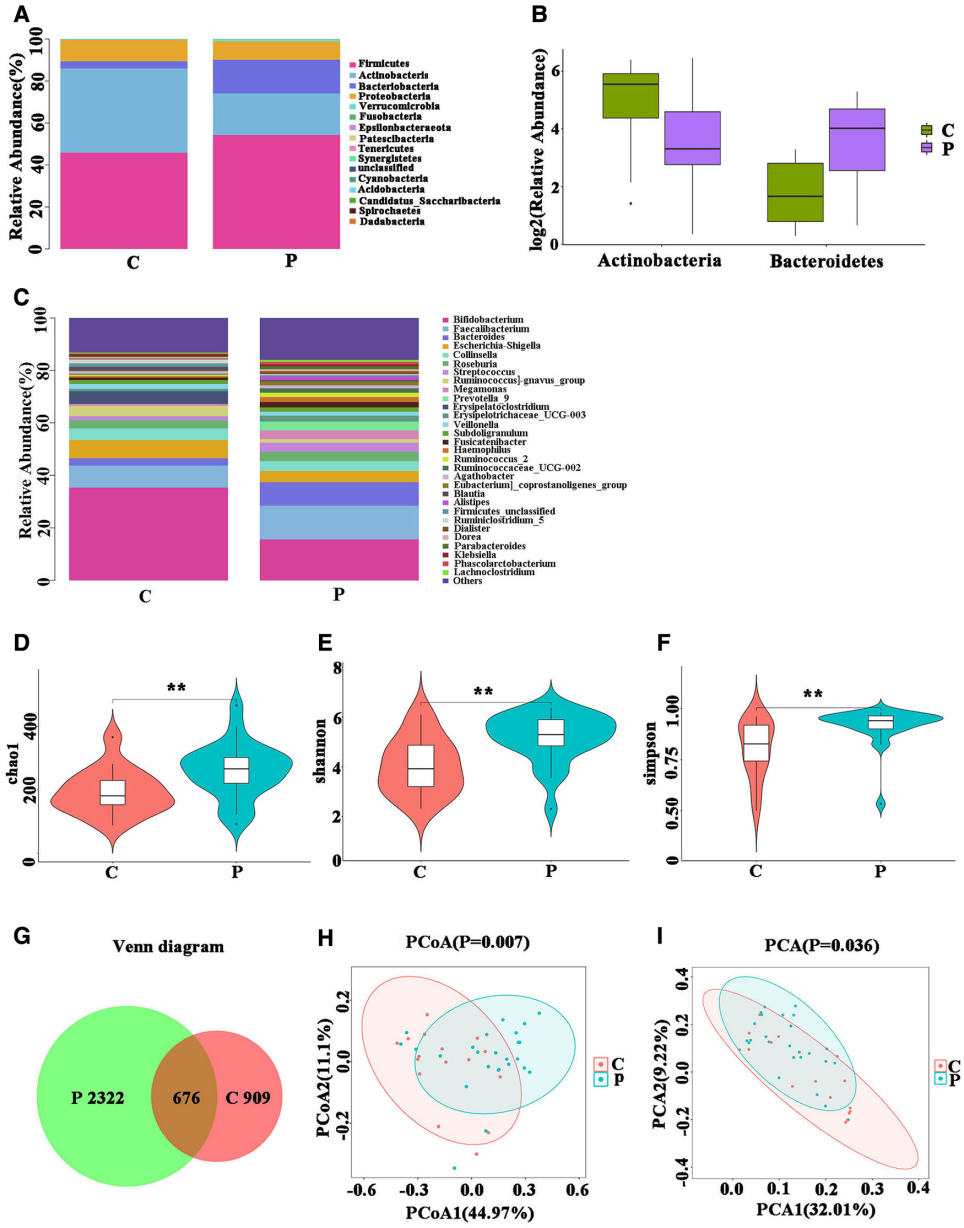
Fecal microbial performance. (**A**) The changes in intestinal bacterial abundance between group P and group C at the phylum level; (**B**) intestinal flora that differed significantly at the phylum level. (**C**) Changes in intestinal bacterial abundance between group P and group C at the genus level. (**D–F**) Analysis of the *α* diversity of Gut microbiota. (**G**) Venn Diagram of Shared Operational Taxonomy Unit (OTU). (**H,I**) Beta diversity analysis of Gut microbiota.

Sixteen phyla were detected in groups P and C. The changes in the abundance of the gut microbiota between the two groups were statistically different. Fourteen phyla were identified in ITP patients, most of which were Firmicutes (54.3%), Actinobacteria (19.79%), Bacteroidetes (16.06%), Proteobacteria (8.75%), whereas 15 phyla were detected in the healthy volunteers: Firmicutes (45.84%), Actinobacteria (40.15%), Bacteroidetes (3.42%), Proteobacteria (10.23%). Compared with those of the controls, the proportion of Firmicutes and Bacteroidetes in patients with ITP were found to be increased, while the proportions of Actinobacteria and Proteobacteria were found to be decreased (Figure 1A), with increase seen in Bacteroides (*P* = 0.00) and the decrease seen in Actinobacteria (*P* = 0.01) being significant (Figure 1B). Next, we evaluated gut microbiota at the genus level and found a significant decrease in Bifidobacterium and Reunions and a significant increase in Bacteroides, a result which was consistent with our findings at the phylum level (Figure 1C).

### 3.3. Diversity analysis of gut microbiota

We analyzed the diversity of gut microbiota linked to ITP. In diversity analyses, the Chao1 index indicates the abundance of species in the community. We found significant differences between the abundances in the two groups (Figure 1D). The Shannon and Simpson indices, which consider both species richness and evenness, indicated that the two groups were statistically different (Figures 1E,F). Statistical results indicated that the richness and diversity of group P were higher than those of group C.

We constructed a Venn diagram which shows the overlapping OTU data of the two groups, to evaluate their shared richness (Figure 1G). The *β* diversity of Gut microbiota, indicated that the overall structure of Gut microbiota of the P group was significantly different from that of the C group. (Figures 1H,I).

The above analysis indicated that there were differences in the bacterial abundance and diversity between the gut microbiota of patients with ITP and healthy controls, and that there were significant differences between the overall structures of the gut microbiota of the two groups.

### 3.4. Variations in gut microbiota distribution among different age groups

**Figure 2:**
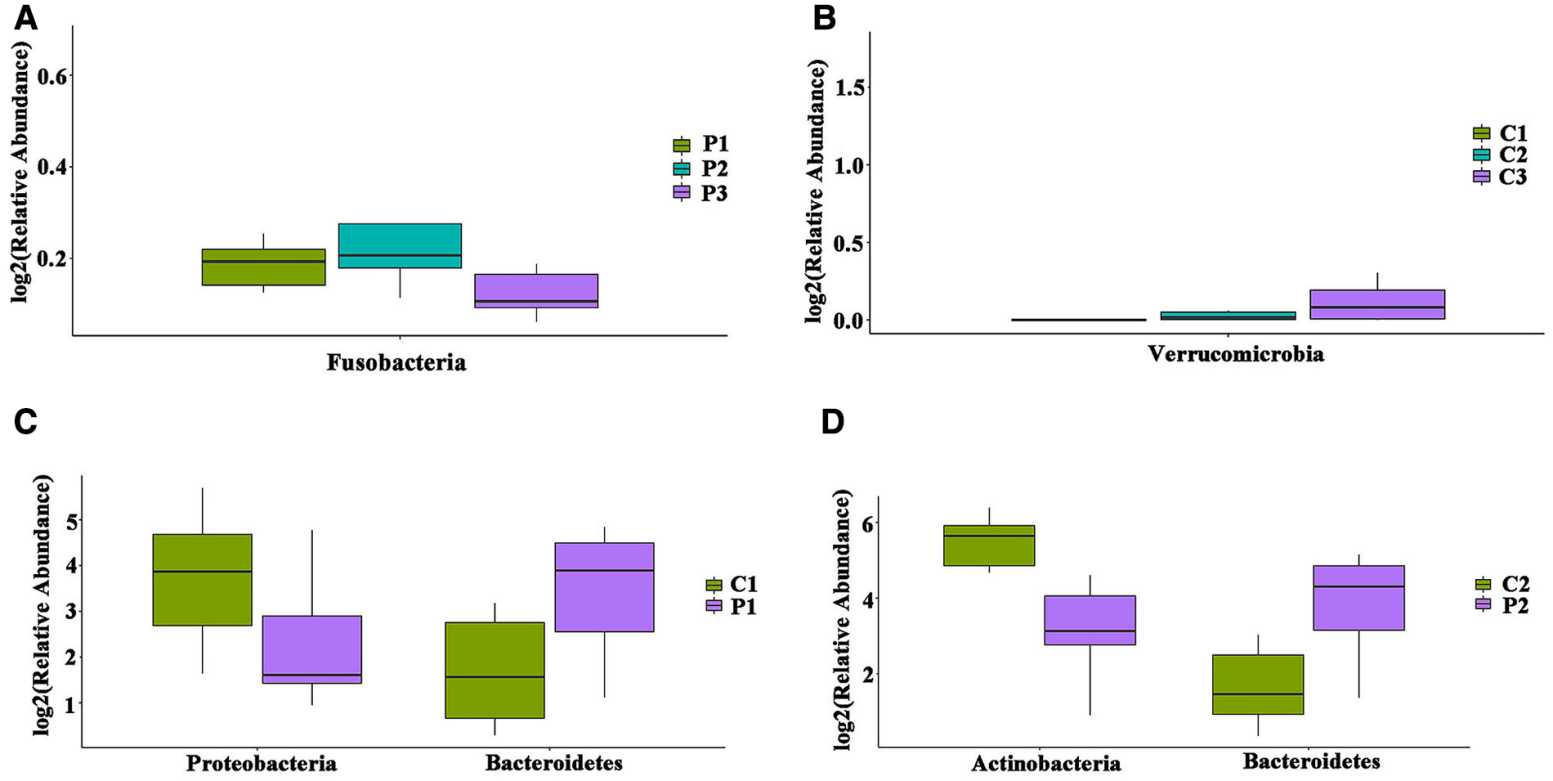
Differences in the distribution of Gut microbiota in different age groups at the phylum level. (**A**) Intestinal flora with significant differences between age groups in P. (**B**) Intestinal flora with significant differences between age groups in C. (**C**) Intestinal flora with differences in P1 and C1; (**D**) intestinal flora with differences in P2 and C2.

We further studied group P to determine whether there were differences in the gut microbiota between the different age groups. Testing the phylum levels of the gut microbiota, the abundance of Fusobacterium in P3 was significantly decreased compared to that in P1 (Figure 2A). Similar changes were observed at the genus level (Supplementary Figures A,B). The abundance of Verrucomicrobia in group C3 was significantly higher than that in group C1 (Figure 2B). Fusobacterium showed age related differences in group P, whereas Verrucomicrobia showed age related differences in group C. The above results indicated that although Fusobacteria and Verrucomicrobia did not show differences between groups P and C, they showed age-based differences within each group.

In order to avoid the interference of age factors on the gut microbiota, we compared the distribution of gut microbiota between same age groups at the phylum level. Compared with the corresponding groups in C, the P1 and P2 groups showed statistically significant differences, where Bacteroidetes was increased, while Proteobacteria was decreased in group P1 (Figure 2C), whereas Bacteroidetes was increased, while Actinobacteria was decreased in group P2 (Figure 2D). At the genus level, Bacteroides was increased (*P* = 0.04), while Pseudomonas was decreased (*P* = 0.01) in group P1 (Supplementary Figure C); whereas Bacteroidetes was increased (*P* = 0.03), while Bifidobacterium was decreased (*P* = 0.02) in group P2 (Supplementary Figure D); and Escherichia-Shigella was increased (*P* < 0.01) in group P3 (Supplementary Figure E).

### 3.5. The abundance level of certain bacteria is related to ITP

**Figure 3:**
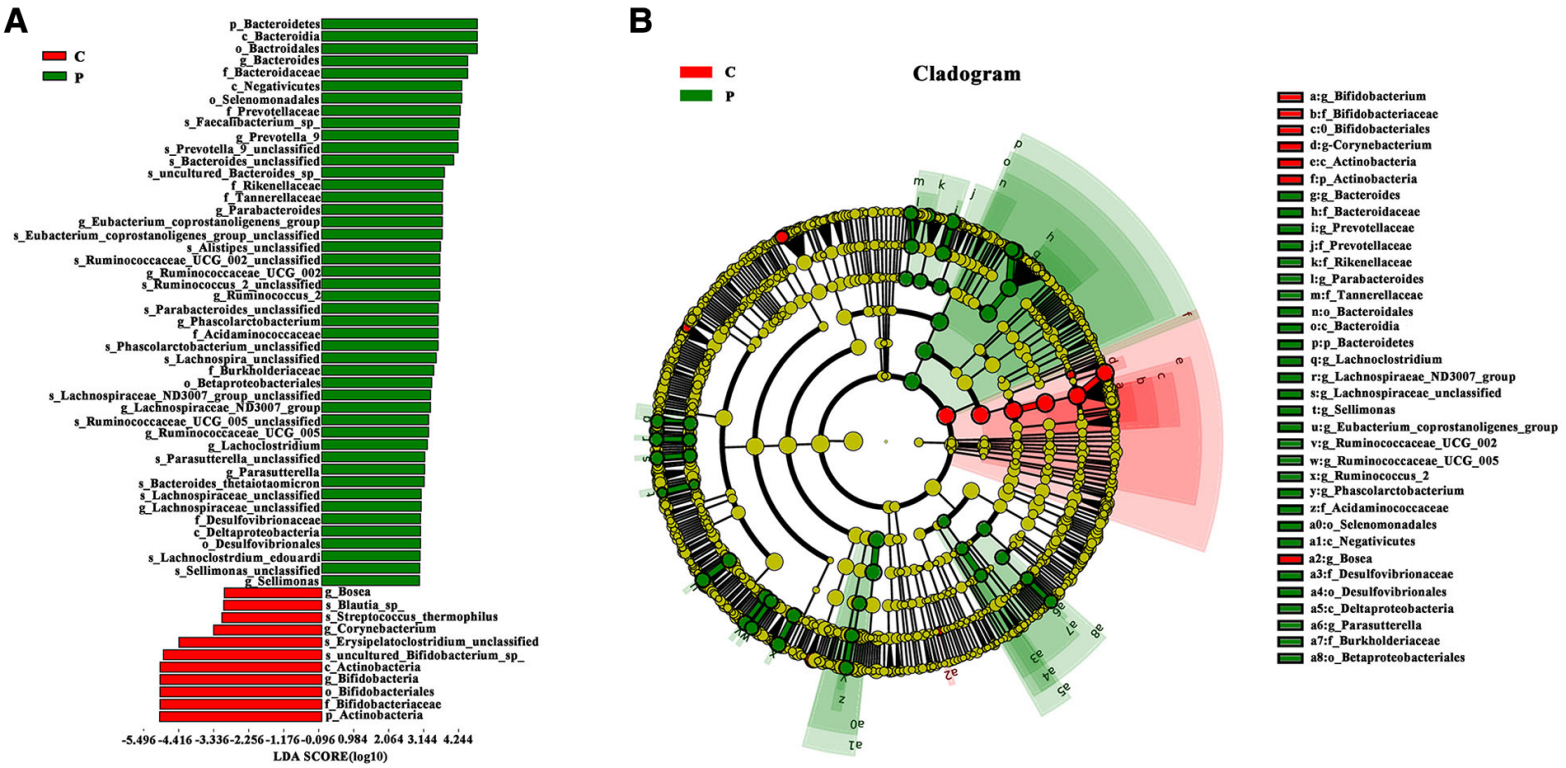
Advanced analysis of gut microbiota. (**A**) The Linear discriminant effect size analysis (LEfSe) corresponding to the most differentially abundant bacterial taxa. The histogram of LDA scores shows the biomarkers showing statistical difference between groups. The red and green bars represented healthy controls and ITP patients’ samples, respectively. The influencing degree of species was expressed by the length of bar in upper histogram. (**B**) In the cladogram, the circle radiated inside-out demonstrated the classification (from phylum to genus). Each small circle at different classification represented taxa and the diameter of circle is proportional to the relative abundance. Species not showing significant differences are colored yellow and biomarkers were colored by different groups. Red and green dots represent the core bacterial populations in respective groups.

To identify bacteria that are specifically associated with ITP, the fecal microbiota compositions of patients with ITP and healthy controls were analyzed using the linear discriminant analysis effect size (LEfSe) method. LEfSe analysis revealed that 46 Escherichia-Shigella bacterial groups were specifically associated with ITP, while 11 bacterial groups were associated with healthy controls (Figures 3A,B). Among them, phylum Bacteroidetes was widespread in ITP patient samples [linear discriminant analysis (LDA)> 2, *P* < 0.05], while control group samples were rich in phylum Actinobacteria (/LDA/>2, *P* < 0.05). Genus Bacteroides was widespread in patients with ITP, while control group samples were rich in genus Bifidobacterium.

### 3.6. Correlation analysis of gut microbiota and platelet count in patients with ITP

Our study indicated that Bacteroidetes and Actinobacteria underwent statistically different changes at the phylum level. On this basis, we evaluated the relationship between the relative abundance of these phyla and platelet counts (PLTs) in patients with ITP and the controls. The results showed that there was no significant correlation between platelet counts (*P* > 0.05). At the same time, we analyzed the time required for PLTs and gut microbiota changes to return to normal levels, but statistical analysis showed no significant correlation (*P* > 0.05).

### 3.7. Correlation analysis of gut microbiota, PLT and IgG in patients with ITP

We analyzed the correlation between antiplatelet antibodies, IgG, IgA, and IgM, and platelets in ITP patients, and found that PLT was significantly negatively correlated with IgG (*P* = 0.021), while there was no significant correlation between PLT and IgM or IgA. We conducted a correlation analysis of antiplatelet antibodies and gut microbiota (Bacteroidetes and Actinobacteria) and found that IgG was significantly positively correlated with Bacteroides (*P* = 0.006); (Table 3).

**Table 3:** Correlation analysis of Gut microbiota, PLT and platelet autoantibodies in patients with ITP.

	Actinobacteria		Bacteroidetes		PLT	
	r	*p*	r	*p*	r	*p*
PLT	−0.191	0.382	−0.164	0.455	/	/
IgG	−0.047	0.852	0.623	0.006	−0.540	0.021
IgA	−0.180	0.475	−0.026	0.918	−0.195	0.438
IgM	−0.304	0.220	−0.124	0.625	0.136	0.592

## 4. Discussion

Current clinical research has revealed a strong connection between gut microbiota and immune-related diseases, such as Graves' disease, autoimmune encephalomyelitis, multiple sclerosis, atopic dermatitis, and rheumatoid arthritis (17–22). Primary immune thrombocytopenia (ITP) is an acquired autoimmune hemorrhagic disease (23). Compared to adults, children with ITP are more likely to recover spontaneously without intervention, and approximately 69% of children with ITP achieve complete remission within 6 months (24). The pathogenesis of primary immune thrombocytopenia is not fully understood. The association between adult primary immune thrombocytopenia and gut microbiota has been studied. Therefore, we studied the changes in the gut microbiota in children with ITP. In this study, we analyzed the gut microbiota of fresh stool samples collected from 25 newly diagnosed ITP children and 16 healthy volunteers. Firmicutes and Actinobacteria have the most significant changes. In addition, there were also differences between the gut microbiotas of different age groups within each of the two main groups. The gut microbiota of patients showed specific changes in diversity and were correlated with antiplatelet antibodies.

We compared the gut microbiota of children with ITP with those of healthy controls and found that, in the former, the proportion of phylum Bacteroidetes was significantly increased while that of phylum Actinobacteria was significantly decreased, compared to the latter (controls). This observation differed from changes observed in the gut microbiota of adult patients with ITP. Liu (11) showed that the proportion of Bacteroidetes in the microbiota of patients with ITP had increased, whereas Zhang (12) found that the presence of Bacteroides in the microbiota of patients with ITP was significantly reduced. We propose that such differences in research results may be associated with sample size. Studies have shown that decreased alpha diversity is a reliable indicator of disease-related malnutrition (25, 26). In adult ITP patients, it was found that the Simpson index was low indicating that alpha diversity had decreased (12); however, our research suggests that the alpha diversity is increased in children with ITP, which is different from that reported for adults, and may need to be confirmed by increasing the sample size further. In addition, we also found statistical differences in *β* diversity, suggesting that the structure of the gut microbiota of ITP patients was different from that of the controls.

The composition of gut microbiota in ITP patients was also different from that of healthy controls at different ages. The within the group differences were consistent with the changes seen in the gut microbiota of the groups, with changes in P1 and P2 reaching statistical significance. The P3 group did not show statistical significance, and considering that this may be related to the number of samples involved, we believe that sample size should be increased further when studying differences between the flora of different age groups and their impact on incidence.

Platelet Associated Immunoglobulin G (PA-IgG) was significantly increased in ITP patients and that the amount of PA-IgG was related to the severity of the disease. There was a clear negative correlation between PA-IgG levels and PLT (27). Megakaryocytes in the bone marrow of patients with ITP exhibit strong binding to IgG (28). IgG and IgM antiplatelet antibodies can be used to diagnose ITP (29). Studies have suggested that detection of PA-IgG may provide a useful predictor of the response to first-line corticosteroid therapy for ITP (30), Our research found that PLT and IgG were significantly negatively correlated, while IgG and phylum Bacteroidetes were significantly positively correlated (*P* < 0.01), suggesting that antiplatelet antibodies may reflect changes in gut microbiota to a certain extent, and also that they may participate in the pathogenesis of ITP disease via IgG. On this basis, they may be used to judge diseases and guide treatment.

Our study was affected by certain limitations. First, this was a single-center study with a small sample size. Second, the patients who were included were all new-onset ITP patients, making it impossible to assess the differences between patients presenting with different disease stages of ITP. Overall, this study found that the gut microbiota of patients with ITP was altered compared to that of healthy individuals. The main characteristics were an increase in the abundance of phylum Bacteroides accompanied by a decline in that of Actinobacteria. There were differences between the gut microbiota of different age groups, as well as in diversity. Furthermore, gut microbiota were correlated with antiplatelet antibodies. We believe that the findings of this study would provide insights into ITP treatment.

## 5. Conclusions

Gut microbiota disorders in children with ITP are characterized by changes in biodiversity and composition. Gut microbiota varied by age group, and a certain correlation exists between gut microbiota and antiplatelet antibodies, which are positively correlated with PA-IgG, indicating that IgG may play a role in the pathogenesis of ITP via gut microbiota.

## Data Availability

The original contributions presented in the study are included in the article/**Supplementary Material**, further inquiries can be directed to the corresponding authors.
