# Supplementary material for: Gut microbiota alterations in children and their relationship with primary immune thrombocytopenia
Source: Front Pediatr. 2023 Jun 21;11:1213607. doi: 10.3389/fped.2023.1213607 (PMC10320726; doi:10.3389/fped.2023.1213607)
Supplement: Supplementary file 1 [file Datasheet1.docx]

Supplementary Material

**Gut microbiota alterations in children and their relationship with primary immune thrombocytopenia**

**Xiangyu Li^1^, Minna Zhang^1^, Le He^1^, Jingfang Zhou^1^, Peng Shen^1^, Weijie Dai^1^, Xiaozhong Yang^1^, Yufang Yuan^2^, Haiyan Zhu^2*^, Honggang Wang^1*^**

^1^ Department of Gastroenterology, The Affiliated Huaian No.1 People’s Hospital of Nanjing Medical University, Huaian 223300, Jiangsu Province, China

^2^ Pediatrician department, The Affiliated Huaian No.1 People’s Hospital of Nanjing Medical University, Huaian 223300, Jiangsu Province, China

*** Correspondence:** Honggang Wang, email:[jgzwhg@njmu.edu.cn](mailto:jgzwhg@njmu.edu.cn); Haiyan Zhu, email:[zhy4600@126.com](mailto:zhy4600@126.com(H-Y).

# Supplementary Figure


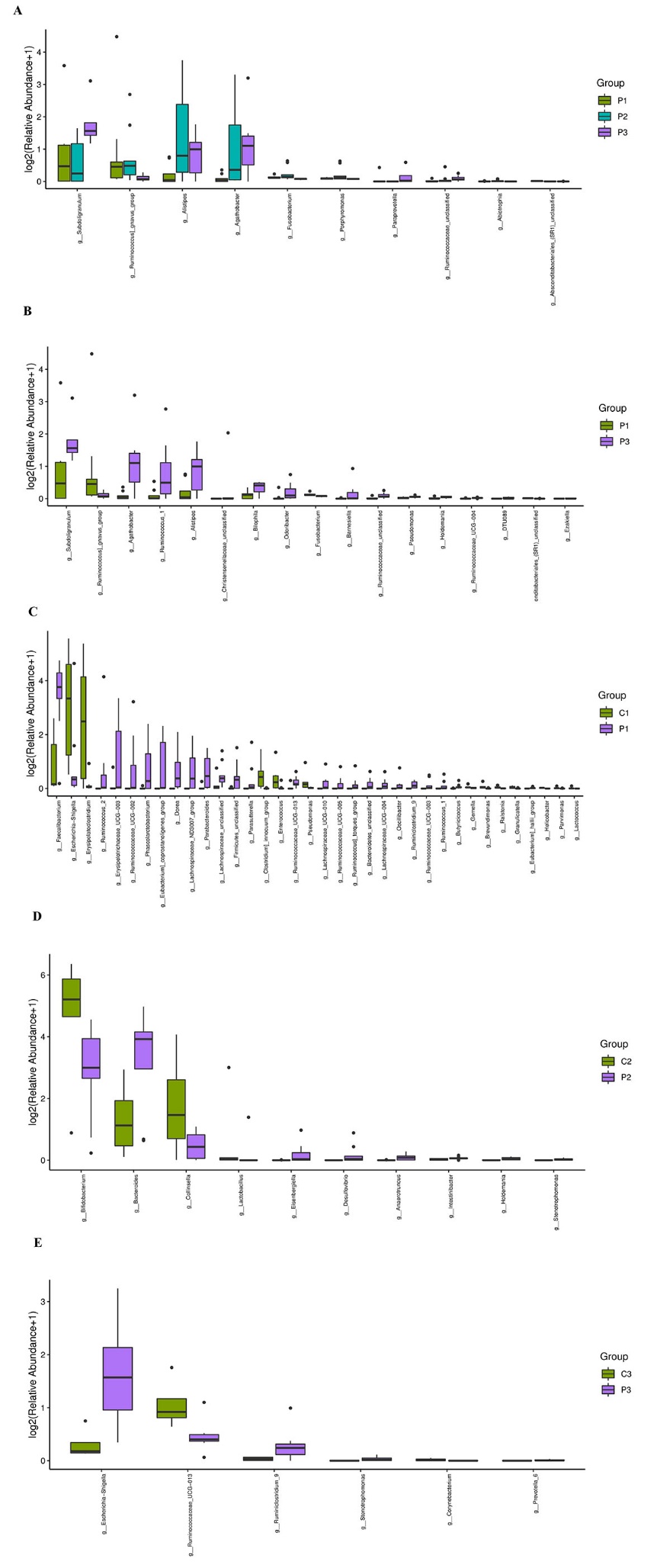


**Supplementary Figure. Differences in the distribution of Gut microbiota in different age groups at the genus level.** (A) Fusobacteria had statistical differences in different age groups of P group. (B) Compared with P1, Fusobacteria in P3 decreased significantly, with statistical differences. (C) the P1 group Bacteroidetes increased, and the Pseudomonas decreased. (D) in P2 group, Bacteroidetes increased, Bifidobactreium decreased. (E) in P3 group, Escherichia-Shigella increased(P＜0.01).
